# Implementation and Evaluation of a Telemedicine Program for Specialty Care in North Carolina Correctional Facilities

**DOI:** 10.1001/jamanetworkopen.2021.21102

**Published:** 2021-08-16

**Authors:** Saif Khairat, Aaron Bohlmann, Erin Wallace, Adnan Lakdawala, Barbara S. Edson, Terri L. Catlett, Spencer D. Dorn

**Affiliations:** 1 Carolina Health Informatics Program, University of North Carolina at Chapel Hill; 2 School of Nursing, University of North Carolina at Chapel Hill; 3 Cecil G. Sheps Center for Health Services Research, University of North Carolina at Chapel Hill; 4 UNC Health, Chapel Hill, North Carolina; 5 Healthcare Administration, North Carolina Department of Public Safety, Raleigh; 6 Department of Medicine, University of North Carolina at Chapel Hill

## Abstract

This cross-sectional study evaluates the implementation of a telemedicine program in North Carolina prisons based on responses from individuals who were incarcerated, health care practitioners, and telepresenters.

## Introduction

More than 1.2 million adults are incarcerated in US prisons.^1^ Although these individuals generally receive primary and urgent care within the prison facility, those who need more specialized care often need to leave the prison facility for health care at a local or regional health care facility, a process that is expensive and logistically challenging and may fragment care and pose security risks. In response to the COVID-19 pandemic, prison facilities necessarily limited movement of individuals who were incarcerated within and between facilities,^2^ restricting their ability to access secondary and tertiary health care. The North Carolina Department of Public Safety, UNC Health, and University of North Carolina School of Medicine responded by expediting the implementation of a law passed in North Carolina to enable individuals who are incarcerated to receive specialty care via telemedicine.^3^ The purpose of this study was to evaluate the implementation of a telemedicine program for specialty care in North Carolina prisons during the COVID-19 pandemic.

## Methods

This cross-sectional study was deemed exempt from review by the University of North Carolina at Chapel Hill institutional review board because the evaluation protocol met the definition of a limited data set under 45 CFR§164.514 (e). The North Carolina Department of Public Safety obtained written informed consent from all patients. A Data Use Agreement was executed between the North Carolina Department of Public Safety and the University of North Carolina at Chapel Hill. Since this research was limited to secondary data use with no contact with practitioners, the University of North Carolina at Chapel Hill institutional review board did not require research consent from practitioners or telepresenters. We followed the Strengthening the Reporting of Observational Studies in Epidemiology (STROBE) reporting guideline.

We conducted a cross-sectional study of the use of telemedicine to deliver secondary and tertiary health care by specialist physicians, nurse practitioners, (NPs), and physician assistants (PAs) to individuals who were incarcerated within 55 North Carolina prison facilities. We procured and implemented telemedicine software and equipment, developed a telemedicine workflow, and trained administrative and nursing staff as telepresenters to schedule and assist with telemedicine visits. Likewise, we designated practitioners in each participating specialty to provide care via telemedicine, reassigned established patients who were incarcerated to those practitioners, built practitioner schedules, developed a scheduling web portal and referral triage process, and trained practitioners to perform telemedicine visits and document these encounters in the prison electronic health record.

Between June 1 and November 30, 2020, we distributed in-prison surveys for 1252 visits and collected 1584 surveys from patients (482 visits; response rate, 38.5%) and telemedicine presenters (739 visits; response rate, 59.0%). Patient race was self-identified and collected to assess whether there were racial disparities in telemedicine satisfaction. Additionally, we collected electronic surveys from practitioners at the end of each telemedicine shift, which covered 3232 visits performed by 60 practitioners (316 telemedicine shifts; 58 practitioners [96.7%] among all participating practitioners completed ≥1 survey).

The survey instrument contained six 5-point scale questions regarding overall satisfaction (all participants), comfort using telemedicine technology (all participants), visit duration (patients only), explanation of treatment plan (patients only), quality of telemedicine call (practitioners and telepresenters), and ability to assess patient condition (practitioners only). We used descriptive statistics to analyze the survey responses. Kruskal-Wallis χ^2^ test was used to compare satisfaction scores between professional roles. *P* values were 2-sided, and statistical significance was set at *P* < .05. Data were analyzed from November 1, 2020, to March 1, 2021, using R statistical software version 1.4.1106 (R Project for Statistical Computing).

## Results

A total of 482 patients (244 patients [50.6%] aged ≥51 years; 424 [88.0%] men; 225 African American individuals [46.7%]) were included. Of 316 practitioners included, 228 (72.7%) were men and 221 (69.9%) were physicians (Table).

**Table.** Patient, Practitioner, and Telepresenter Telemedicine Experience Ratings by Age, Sex, and Race

Characteristic	Participants, No. (%)	Rating, Mean (SD), points^a^			
		Overall satisfaction	Personal comfort	Visit duration or call quality^b^	Treatment explained or ability to assess patient^c^
Total	1584	4.24 (0.98)	4.18 (1.00)	NA	NA
**Patients**					
Total	482 (100)	4.22 (0.98)	4.17 (1.02)	4.21 (0.96)	4.33 (0.93)
Race					
American Indian or Alaska Native	25 (5.2)	4.04 (1.27)	3.96 (1.23)	3.83 (1.27)	3.91 (1.34)
Asian	3 (0.6)	3.67 (1.15)	4.00 (1.00)	3.67 (1.15)	3.67 (1.15)
African American	225 (46.7)	4.14 (1.02)	4.07 (1.09)	4.16 (0.97)	4.31 (0.91)
Native Hawaiian or other Pacific Islander	10 (2.1)	4.40 (0.97)	4.20 (1.23)	4.50 (0.97)	4.50 (0.97)
White	195 (40.5)	4.34(0.93)	4.33 (0.88)	4.32 (0.91)	4.40 (0.88)
Not reported	24 (5.0)	4.20 (0.81)	4.00 (1.00)	4.11 (0.90)	4.26 (0.78)
Sex					
Men	424 (88.0)	4.18 (0.99)	4.12 (1.04)	4.17 (0.98)	4.30 (0.94)
Women	24 (5.0)	4.75 (0.53)	4.79 (0.51)	4.75 (0.53)	4.83 (0.48)
Not reported	34 (7.1)	4.33 (1.04)	4.34 (0.88)	4.21 (0.94)	4.33 (0.94)
Age, y					
18-34	69 (14.3)	3.96 (1.33)	3.99 (1.24)	4.02 (1.25)	4.03 (1.21)
35-50	156 (32.4)	4.31 (0.88)	4.13 (1.04)	4.18 (0.92)	4.38 (0.84)
≥51	244 (50.6)	4.25 (0.95)	4.26 (0.94)	4.29 (0.91)	4.38 (0.88)
Not reported	13 (2.7)	4 (0.81)	3.89 (0.87)	3.67 (0.94)	4 (0.82)
**Practitioners**					
Total	316 (100)	4.06 (1.17)	4.27 (1.09)	4.24 (0.95)	3.97 (1.12)
Sex					
Men	217 (73.1)	4.12 (1.17)	4.37 (0.94)	3.43 (0.92)	4.22 (1.00)
Women	80 (26.9)	3.36(1.10)	3.59 (1.17)	3.96 (0.98)	3.31 (1.18)
Professional title					
Physician	211 (71.5)	3.68 (1.24)	3.92 (1.14)	4.11 (0.99)	3.72 (1.12)
Physician assistant	60 (20.3)	4.63 (0.94)	4.78 (0.88)	4.71 (0.69)	4.73 (0.83)
Nurse practitioner	45 (14.2)	3.76 (0.99)	4.19 (0.68)	3.79 (0.64)	3.71 (0.85)
**Telepresenters**					
Total	786 (100)	4.29 (0.93)	4.17 (0.96)	4.34 (0.84)	NA
Telehealth medium					
Video	743 (94.5)	4.35 (0.89)	4.20 (0.95)	4.38 (0.81)	NA
Telephone	22 (2.8)	3.67 (1.06)	3.67 (1.06)	3.83 (0.89)	NA
Not reported	21 (2.7)	3.67(1.28)	3.84 (1.02)	3.95 (1.31)	NA

Abbreviation: NA, not applicable.

^a^ Survey metrics used a 5-point scale, with 1 indicating poor; 2, fair; 3, good; 4, very good; and 5, excellent.

^b^ Patients reported on visit duration, and practitioners and telepresenters reported on call quality.

^c^ Patients reported on explanation of treatment, and practitioners reported on the ability to assess the patient.

Among all patients, 453 patients (94.0%) reported a positive overall telemedicine experience (Figure, A-B). The aspect of telemedicine with the highest patient rating was practitioner communication regarding the treatment plan (mean [SD] rating, 4.33 [0.93] points), and personal comfort using telemedicine was the lowest rated aspect (mean [SD] rating, 4.17 [1.02] points) (Table).

**Figure.**
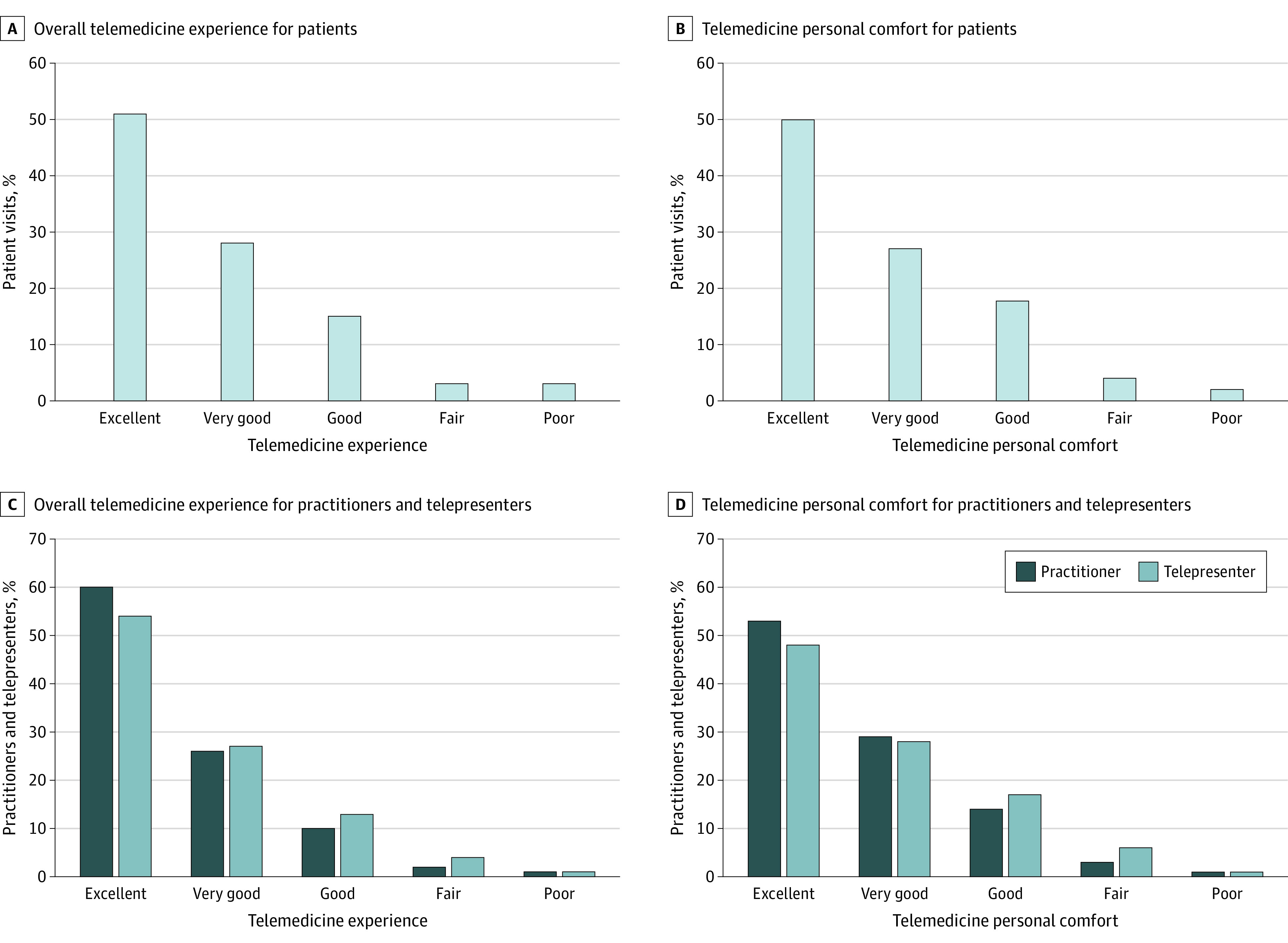
Patient, Practitioner, and Telepresenter Satisfaction Survey Responses Regarding Overall Telemedicine Experience and Comfort Level

Among all practitioners, 272 practitioners (86.1%) were satisfied with the telemedicine visits and 284 practitioners (89.9%) felt comfortable using telemedicine (Figure, C-D). The aspect of telemedicine with the highest practitioner rating was comfort using telemedicine (mean [SD rating, 4.27 [1.09] points), and ability to assess patient condition was rated the lowest (mean [SD] rating, 3.28 [0.86] points). Physicians were significantly less satisfied with the overall telemedicine experience (mean [SD] rating, 3.68 [1.24] points) compared with PAs (mean [SD] rating, 4.63 [0.94] points) and NPs (mean [SD] rating, 3.76 [0.99] points) (χ^2^_4_ = 20.86; *P* < .001).

Among all telepresenters, 739 telepresenters (94.0%) were satisfied using telemedicine and 731 telepresenters (93.0%) were comfortable using telemedicine (Figure, C-D). The aspect of telemedicine with the highest rating among telepresenters was telemedicine call quality (mean [SD] rating, 4.34 [0.84] points), and comfort using telemecine was rated the lowest (mean [SD] rating, 4.17 [0.96] points).

## Discussion

This cross-sectional study evaluated the implementation of a telemedicine program in North Carolina prisons based on survey responses from individuals who were incarcerated, health care practitioners, and telepresenters. In the face of the COVID-19 pandemic, we rapidly implemented a telemedicine program across the North Carolina prison system. We found that telemedicine was well received by patients, nursing staff, and practitioners. There were differences in satisfaction rating based on professional roles. This supports previously reported rates of satisfaction among patients, practitioners, and telepresenters.^4,5^ The telemedicine program was critical for maintaining care access and ensuring care continuity during the pandemic.^6^

This study has limitations. There were no pre–COVID-19 satisfaction data to serve as a baseline measure. Additionally, social desirability bias, such as lack of empowerment among individuals who are incarcerated, was not accounted for.

The findings of this cross-sectional study emphasize that populations at increased risk, including people who are incarcerated, along with health care practitioners and nursing staff, found merit in using telemedicine to continue specialty care during the pandemic.
